# The prognostic value of Epstein−Barr virus infection in Hodgkin lymphoma: A systematic review and meta-analysis

**DOI:** 10.3389/fonc.2022.1034398

**Published:** 2022-10-27

**Authors:** Jianyu Hu, Xue Zhang, Huan Tao, Yongqian Jia

**Affiliations:** Department of Hematology, West China Hospital, Sichuan University, Chengdu, China

**Keywords:** Epstein – Barr virus, Hodking's lymphoma, Meta - analysis, prognosis, Virus infection

## Abstract

**Introduction:**

Epstein−Barr virus (EBV) contributes significantly to the development and occurrence of B-cell lymphomas. However, the association between EBV infection status and clinical outcomes in Hodgkin lymphoma (HL) patients has long been controversial. Therefore, we aimed to estimate the prognostic significance of EBV infection in HL survival.

**Methods:**

We searched PubMed, Embase, Web of Science, and the Cochrane Library for relevant cohort studies from the date of their inception to February 20, 2022. Hazard ratios (HRs) and 95% confidence intervals (CIs) for overall survival (OS), Failure-free survival (FFS), Progression-free survival (PFS), Event-free survival (EFS) and disease-specific survival (DSS) were extracted from the studies or calculated. Subgroup analyses were conducted independently on the five survival outcomes to investigate the source of heterogeneity.

**Results:**

A total of 42 qualified studies involving 9570 patients were identified in our meta-analysis. There was an association between EBV positivity and significantly poorer OS (HR=1.443, 95% CI: 1.250-1.666) and DSS (HR=2.312, 95% CI: 1.799-2.972). However, the presence of EBV in HL showed no effect on FFS, PFS or EFS. In subgroup analyses of OS, DSS and FFS stratified by age groups, EBV positivity was associated with poorer prognosis in elderly patients. Meanwhile, in children and adolescents with EBV-positive HL, we also observed a trend toward a better prognosis, though the results were not statistically significant.

**Conclusions:**

EBV-positive status is associated with poor OS and DSS in HL patients. EBV infection should therefore be considered a valuable prognostic marker and risk-stratifying factor in HL, especially in older patients.

**Systematic Review Registration:**

https://www.crd.york.ac.uk/PROSPERO/, identifier CRD42022328708.

## Introduction

Hodgkin lymphoma (HL) is a malignant neoplasm derived from B lymphocytes, accounting for approximately 10% of all human lymphomas (1). HL is one of the most frequent neoplasms in young individuals aged 20 to 40 years, accounting for nearly one-third of all new diagnoses (2). After the advent of combination chemotherapy, HL is now a highly curable malignancy. Optimal treatment selected according to standard staging has led to a cure rate exceeding 90% for limited stage disease and 80% for advanced disease as the norm (3). Nevertheless, the implication behind this rather impressive success rate is inevitability over- and undertreatment of at least 10-20% of patients in all stages of the disease. The challenge under such circumstances is to maximize cure rates while minimizing long-term toxicity, such as the induction of a second malignancy, dysplasia, or cardiac dysfunction (4, 5). Therefore, the identification of factors indicating different survival outcomes is critical in guiding risk-adapted therapy for HL.

Currently, commonly used prognostic systems for HL are based mainly on clinical parameters such as Ann Arbor staging and tumor size (6). Clearly, it is necessary to improve the traditional prognostic factors in combination with immunological, biological, and functional imaging data (7). Research on immunohistochemical markers for HL prognosis is currently ongoing, with studies in which the expression of the anti-apoptotic protein B-cell lymphoma-2 (Bcl2), the tumor suppressor protein p53 and topoisomerase IIα are associated with poorer prognosis (8, 9). It is accepted that EBV has transforming potential and that latent infections contribute to the pathogenesis of HL (10). EBV-positive HL is defined as the presence of EBV in tumor cells, not in bystander reactive lymphocytes (11). Currently, EBV-encoded mRNA (EBER) *in situ* hybridization (ISH) is considered to be the “gold standard” for EBV status. Meanwhile, some studies have shown that immunohistochemistry with LMP-1 antibodies can also reliably indicate EBV infection in HL (12, 13).

To date, a large number of studies have reported the correlation between Epstein−Barr virus (EBV) infection and the prognosis of HL, but the results of the studies have been inconsistent (positive, negative or no association) (11, 14–54). The differences in the results of the studies may be explained by different population distributions, patient selection, statistical analysis techniques and outcome measures. Therefore, we conducted a meta-analysis of all eligible published studies to quantify the prognostic value of EBV infection in HL patients.

## Materials and methods

We followed the PRISMA Statement guidelines to conduct and report this systematic review and meta-analysis (55). The study was registered in PROSPERO (Record Number CRD42022328708).

### Literature search

We systematically searched PubMed, Embase, Web of Science and the Cochrane library for articles published from the date of their inception to February 20, 2022. We identified studies by using the following terms: (“Epstein−Barr Virus Infections” or “EBV Infections” or “Epstein−Barr Virus” or “Human Herpes Virus 4 Infections” or “HHV 4”) and (“Hodgkin Disease” or “Hodgkin Lymphoma” or “Hodgkin’s Disease” or “Hodgkin’s Granuloma”) and (“prognosis” or “prognostic factor” or “survival”). The reference lists of the identified articles were also searched manually to ensure that no studies were overlooked.

### Study selection

Two investigators (J. Y. Hu, X. Zhang) independently screened each study based on titles and abstracts. When the studies met our inclusion criteria, the full text of the articles was retrieved. We resolved disagreements through discussions or negotiations with a third investigator (H. Tao). Studies that met the following criteria were included: (1) discussed the prognosis of EBV infection in HL whose infection status was detected by EBER *in situ* hybridization and/or LMP-1 immunohistochemistry; (2) outcomes were survival-related; (3) sufficient survival data were provided; (4) articles were published in English; and (5) cohort design. If the same author or institution published multiple articles, we selected the most informative article.

Studies were excluded if (1) they were reviews, letters, case reports, conference abstracts, or unpublished articles; (2) study subjects were animals; or (3) the study population was human immunodeficiency virus-associated lymphoma.

### Data extraction and outcomes

The data we extracted from selected articles included the following: (1) baseline characteristics (first author, publication year, country, number of patients, median/mean age, histology, etc.); (2) EBV detection method and EBV status; (3) survival outcomes (including overall survival [OS], failure-free survival [FFS], progression-free survival [PFS], event-free survival [EFS], disease-specific survival [DSS]), definitions of the five survival endpoints are summarized in **Table S1**; and (4) statistical evaluations, including Cox regression analysis hazard ratios (HRs), 95% confidence intervals (CIs), and P values. When HR and 95% CIs were absent from the original article, we used the software designed by Tierney et al. (56) to indirectly estimate from Kaplan−Meier curve.

### Quality assessment

The quality of each study was assessed independently by two investigators (J. Y. Hu, X. Zhang) using the Newcastle−Ottawa Scale (NOS) (57). This scale is an eight-item instrument used to assess the selection of participants, study comparability, and ascertainment of the outcome. The NOS scores ranged from 0 to 9, and high-quality studies were defined if the score was more than 6.

### Statistical analysis

We used the HRs and corresponding 95% CIs to investigate the associations between EBV infection and HL survival outcomes (OS, FFS, PFS, EFS and DSS). For a more accurate estimation of the effect of EBV infection, we selected the results of the multivariate model when both multivariate and univariate Cox regression analyses were reported in the same article. Heterogeneity was assessed by the Cochran’s Q test and I^2^ index, which describes the percentage of total variation across studies that is due to heterogeneity rather than chance (58). Statistically significant heterogeneity was defined as I^2^ statistic>50% and/or P value < 0.10 of Cochran’s Q test. When I^2^>50% and/or P<0.10, the random-effects model was used to estimate pooled HRs (59); otherwise, a fixed-effects model was used (60). To explore a potential source of heterogeneity, subgroup analyses were conducted based on variables including continent, histologic subtype, age, detection method, and whether a multivariate or univariate Cox regression was used. Sensitivity analyses were performed to assess the stability of pooled HR by sequentially excluding each study. Publication bias was evaluated by visual inspection of the symmetry of the funnel plot and assessment with Begg’s and Egger’s tests (P<0.05 was deemed strong publication bias) (61). All statistical analyses were performed using Stata Version 15.1. (Stata, College Station, TX, USA), and P<0.05 was considered statistically significant.

## Result

### Search results

**Figure 1:**
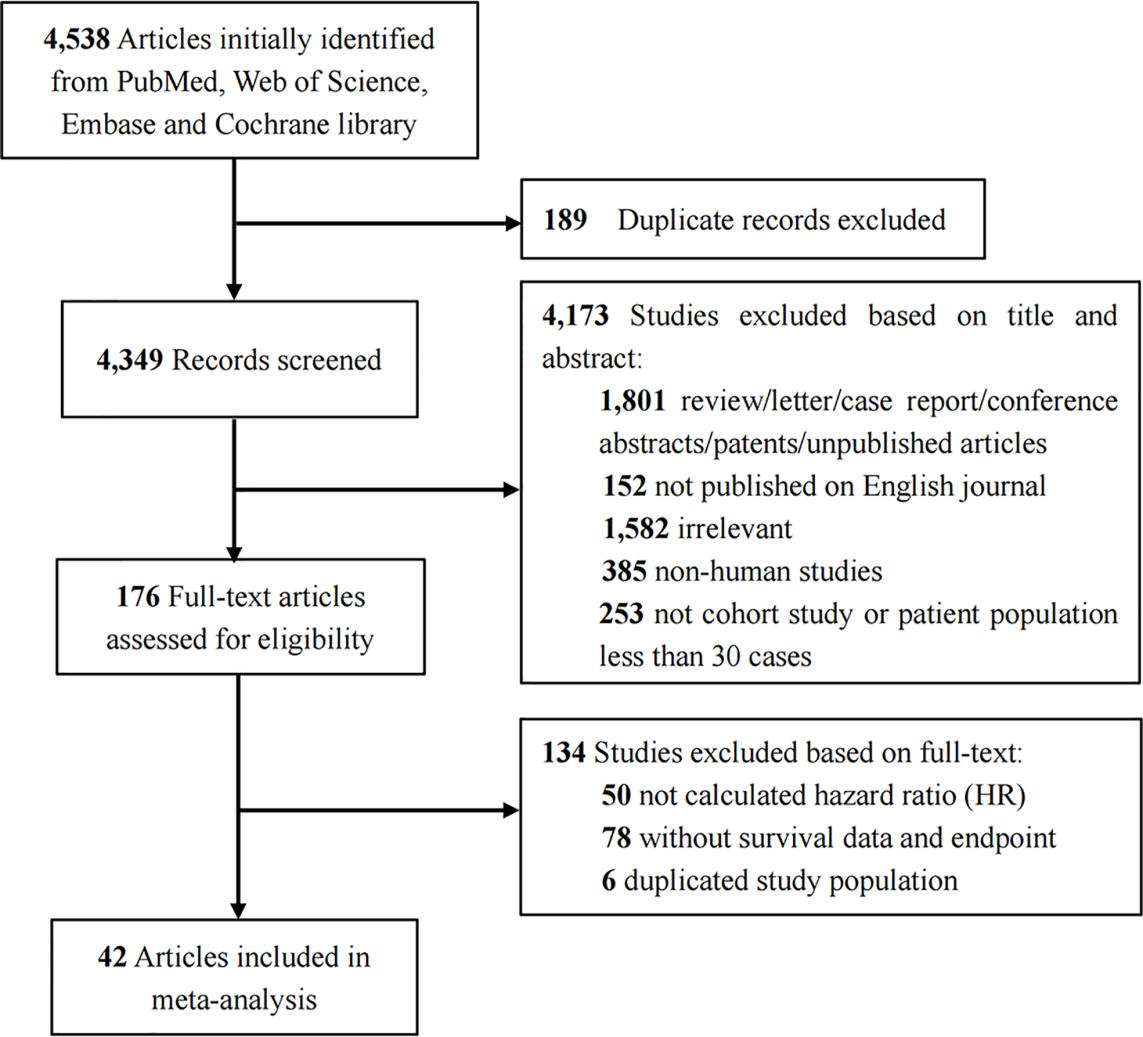
Flow diagram for selection of studies.

**Figure 1** illustrates a flowchart describing the study inclusion process. We initially identified 4538 articles. After the removal of duplicates and screening of titles and abstracts, the full text of the 176 potentially qualified articles was reviewed. Finally, after excluding those with a duplicated study population (n=6), nonsurvival analysis data (n=78) and unable to obtain HR (n=50), 42 articles (11, 14–54) studying 9570 patients were included in our meta-analysis.

### Study characteristics and quality assessment

**Table 1** shows a summary of the characteristics of the 42 included studies, most of which were retrospective cohort studies. The studies were conducted in Asia (37.7%), Europe (33.3%), North America (6.6%), South America (8.8%), Australia (2.2%) and Africa (6.6%) and published between 1997 and 2022. The sample size per study ranged from 47 to 922. The reported mean or median age for studies differed widely; five studies only included patients younger than 18 years old (16, 30, 33, 41, 48), and one study only included the elderly (20). Thirty studies (11, 14, 15, 17, 18, 20–25, 28–30, 32–36, 38, 41, 44–46, 48–51, 53, 54) reported the median or mean follow-up time, ranging from 25 to 130 months. In terms of methodological quality, all included studies scored more than six stars on the NOS. Details of the risk of bias assessment are shown in Additional file: **Table S2**.

**Table 1:** Main characteristics of the included studies.

First author	Year	Country	Inclusion period	N	Median/Mean age (range)	Histology	Median follow-up (Month)	Detection method	EBV+/EBV-	Data source	Data extraction	Outcome	NOS
Yang, L. Q.	2022	China	2010-2020	187	26 (2-82)	HL	48	EBER	106/81	Univariate	K-M curve	OS	7
Wang, C.	2021	China	2012-2019	134	31 (5-74)	HL	56.8^a^	EBER	62/72	Univariate	K-M curve	OS/FFS	8
Santisteban-E, A.	2021	Spain	2009-2020	88	39 (19-82)	cHL	NR	LMP1	36/52	Univariate	K-M curve	OS/PFS	7
Qin, J. Q.	2021	China	2013-2019	96	32 (12-79)	HL	25	EBER	22/40	Multivariate	Direct	OS/PFS	8
Antel, K.	2021	South Africa	2004-2018	77	31 (25-43)	HL	51	EBER/LMP1	39/38	Univariate	K-M curve	OS/PFS	8
Werner, L.	2020	Germany	1991-2007	76	40 (4-84)	HL	80.4^a^	EBER/LMP1	26/50	Univariate	K-M curve	OS	8
Cheriyalinkal P, B.	2020	India	2013-2018	189	NR (≤15)	cHL	29	LMP1	160/29	Multivariate	Direct	OS/EFS	7
Wang, C.	2018	China	2004-2013	86	31.5 (7-82)	HL	NR	EBER	37/49	Univariate	Direct	OS/PFS	8
Koh, Y. W.	2018	South Korea	1990-2016	135	37 (15-78)	cHL	58.92^a^	EBER	50/85	Univariate	Direct	OS	8
Hollander, P.	2018	Sweden and Denmark	1999-2002	459	NR (18-74)	cHL	154.8^a^	EBER/LMP1	122/319	Multivariate	Direct	OS	8
Myriam, B. D.	2017	Tunisia	1998-2012	131	26 (4-83)	cHL	40	EBER	62/69	Univariate	K-M curve	OS/EFS	8
Chang, K. C.^b^	2017	Taiwan	1985-2006	104	39 (6-78)	HL	NR	EBER	48/52	Multivariate	Direct	OS	8
								LMP1	34/53				
Park, J. H.	2016	South Korea	2007-2013	70	39 (14–77)	cHL	NR	EBER	32/38	Univariate	K-M curve	OS/EFS	8
Tanyildiz, H. G.	2015	Turkey	1997-2012	58	11 (3-16)	HL	55	LMP1	20/38	Univariate	K-M curve	OS/FFS	8
Paydas, S.	2015	Turkey	NR	87	35.3 (15-71)	HL	NR	EBER	40/47	Univariate	K-M curve	OS	7
Elsayed, A. A.	2014	Japan	1981-2007	389	48 (4-89)	cHL	NR	EBER	173/216	Multivariate	Direct	OS/PFS	9
Koh, Y. W.	2013	South Korea	1990-2011	167	35 (6-77)	cHL	75.6	EBER	66/101	Univariate	Direct	OS/EFS	8
Kanakry, J. A.	2013	USA	1999-2006	794	32 (16-83)	HL	NR	EBER	51/264	Univariate	Direct	FFS	8
Koh, Y. W.^c^	2012	South Korea	1990-2009	159	32 (4-77)	HL	70	EBER	55/104	Univariate	Direct	DSS	8
Kamper, Peter	2011	Denmark	1990-2007	288	37 (6-86)	cHL	84	EBER/LMP1	95/193	Univariate	Direct	OS/EFS	8
Souza, E. M.	2010	Brazil	1994-2004	97	30 (18-75)	cHL	80	EBER/LMP1	51/46	Univariate	K-M curve	OS/EFS	8
Barros, M. H.	2010	Brazil	1999-2006	104	14 (3–18)	cHL	68	EBER/LMP1	43/55	Univariate	K-M curve	EFS	7
Diepstra, A.	2009	Netherlands	1989-2000	412	35 (7-91)	cHL	85.2 ^a^	EBER	141/271	Multivariate	Direct	FFS	8
Chetaille, B.	2009	France	NR	146	NR	cHL	NR	EBER/LMP1	30/115	Univariate	Direct	OS/EFS	7
Chabay, P. A. ^d^	2008	Argentina	1990-2005	111	8 (2-18)	HL	76	EBER/LMP1	60/51	Univariate	K-M curve	EFS	8
Chabay, P. A.^d^	2008	Brazil	1998-2003	65	14 (3-18)	HL	38	EBER/LMP1	31/34	Univariate	K-M curve	EFS	8
Keresztes, K.	2006	Hungary	NR	109	31 (3-74)	HL	83	LMP1	47/62	Univariate	K-M curve	OS/EFS	7
Jarrett, R. F.	2006	United Kingdom	1993-1997	437	NR (16-74)	cHL	93	EBER	145/292	Univariate	K-M curve	OS/DSS	8
Asano, N.	2006	Japan	NR	324	48 (4-89)	cHL	NR	EBER	149/165	Univariate	Direct	DSS	7
Al-Kuraya, K.	2006	Saudi Arabia	1991-2002	141	NR	HL	NR	EBER/LMP1	24/55	Univariate	K-M curve	OS	6
Keegan, T. H.^e^	2005	USA	1988-1997	922	NR	cHL	97	EBER/LMP1	246/676	Multivariate	Direct	OS/DSS	8
Claviez, A.	2005	Germany	1990-2001	842	13.7 (2.2-20.2)	HL	58.5	LMP1	263/579	Univariate	K-M curve	OS/FFS	8
Krugmann, J.	2003	Austria	1974-1999	119	37.6 (14-83)	cHL	122	LMP1	31/88	Univariate	K-M curve	OS/FFS	8
Herling, M.	2003	USA, Italy, Greece	1984-2000	577	30 (NR)	cHL	65	LMP1	61/242	Univariate	K-M curve	OS/FFS	8
Flavell, K. J.	2003	UK	1983-1996	273	NR	HL	60	EBER	78/195	Multivariate	Direct	FFS	7
Stark, G. L.	2002	UK	1991–1998	102	70 (60–91)	HL	63	LMP1	24/46	Univariate	K-M curve	DSS	8
Glavina-D, M.	2001	Croatia	1980-1990	100	40 (13–84)	HL	NR	LMP1	26/74	Univariate	K-M curve	OS	7
Clarke, C. A.	2001	USA	1988-1994	311	NR (19-79)	HL	73	LMP1	53/258	Univariate	K-M curve	OS	7
Naresh, K. N.	2000	India	1984-1988	110	22 (4-61)	cHL	57	EBER/LMP1	86/24	Univariate	K-M curve	OS	6
Engel, M.	2000	South Africa	NR	47	8 (3-14)	HL	NR	EBER/LMP1	24/12	Univariate	K-M curve	OS	7
Murray, P. G.	1999	UK	1992-1996	190	33 (22-49)	HL	86	EBER/LMP1	51/139	Univariate	K-M curve	OS/FFS	6
Enblad, G.	1999	Sweden	1985-1988	117	45 (11-87)	HL	130	EBER/LMP1	32/85	Univariate	K-M curve	DSS	7
Morente, M. M.	1997	Spain	NR	140	37.2 (5-83)	HL	65	LMP1	72/68	Multivariate	Direct	OS	8

a Mean follow-up time.

b For article written by Chang, K. C. et al., two detection methods were used to define the EBV infection status and the HR were given respectively.

c Koh, Y. W. et al. had another article published in 2013 with similar study population, but this article included for one more endpoint.

d The same article, two patient groups gave HR values respectively.

e For article written by Keegan, T. H., HR values of the total population were not given, and were divided into three age groups.

EBV, Epstein–Barr virus; HL, Hodgkin’s lymphoma; cHL, classical Hodgkin lymphoma; HR, hazard ratios; NOS, Newcastle-Ottawa Scale; EBER, Epstein–Barr virus-encoded small RNA; LMP1, latent membrane protein-1; OS, Overall survival; FFS, Failure-free survival; PFS, Progression-free survival; EFS, Event-free survival; DSS, Disease-specific survival; K-M curve, Kaplan-Meier curve; NR, Not reported.

### Meta-analysis results

#### Overall survival

**Figure 2:**
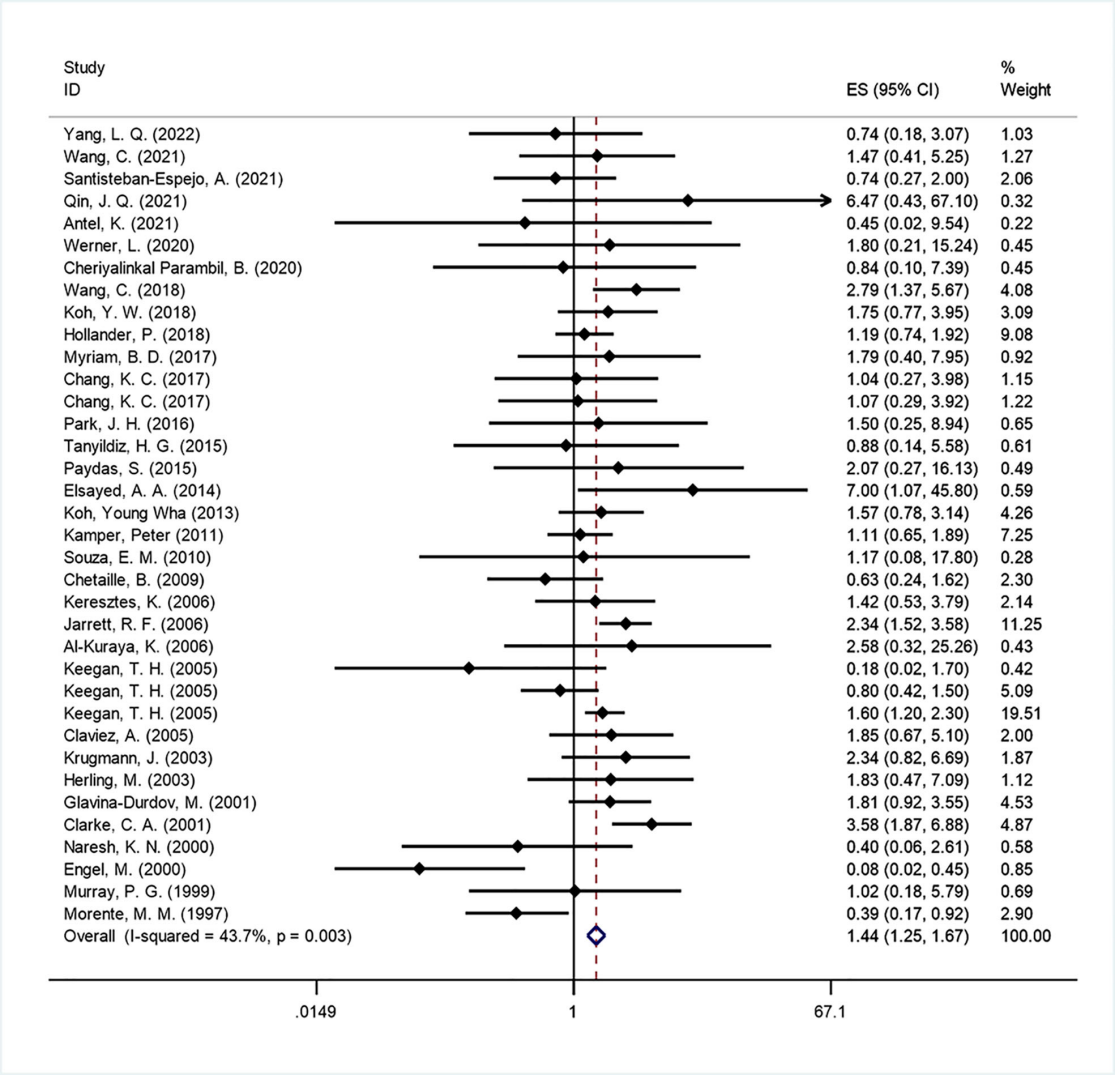
Forest plot of the hazard rations for overall survival (OS) between patients with EBV-positive and EBV-negative HL.

Thirty-three studies (11, 14, 16–19, 22–26, 28, 29, 31, 34, 35, 38–54) (corresponding to 36 sets) were included to analyze the impact of EBV infection on OS. Our meta-analysis showed that EBV positivity in HL was correlated with unfavorable outcomes for OS (HR=1.443, 95% CI: 1.250-1.666, P<0.001; **Figure 2**). Moderate heterogeneity was found across the studies (I^2 =^ 43.7%, P=0.003) by employing a fixed effects model. Therefore, to explain the heterogeneity, we conducted subgroup analyses according to continents, histology, age groups, detection method, data source and data extraction (**Table 2**). In the subgroup analysis by disease distribution on six continents, the African subgroup showed that EBV-positive patients had a borderline better OS (HR=0.408, 95% CI: 0.147-1.129; P =0.084). For age distribution, some articles (16, 18, 25, 28, 41, 48, 50, 52) had sufficient age-stratified survival data, so we combined their HR by a random effects model (I^2 =^ 62.9%, P=0.003), which was 1.080 (95% CI: 0.657-1.776; P=0.762). In the subgroup of children and adolescents, the pooled HR showed that EBV positivity in HL was correlated with a favorable outcome for OS (HR=0.296, 95% CI: 0.085-1.034, P=0.056), while a significantly poorer OS was associated with EBV positivity in studies covering older adults (HR=1.905, 95% CI: 1.380–2.629; P<0.001). This may partly explain the heterogeneity observed when examining EBV infection as a prognostic factor in HL patients.

**Table 2:** Subgroup analysis of relationship between EBV infection and OS/FFS/PFS/EFS/DSS.

Outcome	Subgroup	Data set (n)	Patients (n)	Model	HR (95%CI)	P	Heterogeneity	
							I^2^	P_h_
**OS**	**ALL**	36	7213	Fixed	1.443 (1.250-1.666)	<0.001	43.7%	0.003
	**Continent**			Fixed				
	Asia	15	2057		1.658 (1.205-2.282)	0.002	0	0.696
	Europe	11	2875		1.321 (1.065-1.638)	0.011	51.4%	0.024
	Africa	3	255		0.408 (0.147-1.129)	0.084	74.9%	0.019
	South America	1	97		1.170 (0.078-17.452)	0.909	–	–
	North America	4	1233		1.572 (1.209-2.045)	0.001	78.8%	0.003
	Australia	1	119		2.340 (0.819-6.684)	0.112	–	–
	Unclassified	1	577		1.830 (0.471-7.108)	0.383	–	–
	**Histology**							
	HL	18	2889		1.501 (1.151-1.958)	0.003	55.1%	0.003
	cHL	18	4324		1.420 (1.197-1.684)	<0.001	29.7%	0.114
	**Detection method**			Fixed				
	EBER	12	1606		2.024 (1.551-2.642)	<0.001	0	0.749
	LMP1	11	2409		1.506 (1.121-2.022)	0.007	51.6%	0.024
	EBER/LMP1	13	2363		1.147 (0.930-1.414)	0.200	47.9%	0.027
	**Data source**			Fixed				
	Univariate	27	4247		1.599 (1.328-1.926)	<0.001	39.2%	0.021
	Multivariate	9	2131		1.240 (0.989-1.555)	0.062	51.7%	0.035
	**Data extraction**			Fixed				
	K-M curve	21	3389		1.740 (1.380-2.195)	<0.001	34.6%	0.061
	Direct	15	2989		1.285 (1.070-1.543)	0.007	49.2%	0.016
	**Age subgroups**	11		Random	1.080 (0.657-1.776)	0.762	62.9%	0.003
	children and adolescent	4	330		0.296 (0.085-1.034)	0.056	41.4%	0.163
	young adults	4	995		0.882 (0.518-1.500)	0.642	0	0.882
	older adults	3	480		1.905 (1.380-2.629)	<0.001	23%	0.273
**FFS**	**ALL**	9	3399	Fixed	1.030 (0.832-1.274)	0.788	0	0.556
	**Detection method**			Fixed				
	EBER	4	685		0.961 (0.723-1.277)	0.785	35.7%	0.198
	LMP1	4	1538		1.158 (0.821-1.632)	0.403	0	0.703
	EBER/LMP1	1	190		0.910 (0.361-2.295)	0.842	–	–
	**Data source**			Fixed				
	Univariate	7	1728		1.168 (0.891-1.529)	0.261	0	0.920
	Multivariate	2	685		0.836 (0.591-1.183)	0.312	61.7%	0.106
	**Data extraction**			Fixed				
	K-M curve	6	1728		1.158 (0.853-1.573)	0.346	0	0.852
	Direct	3	685		0.921 (0.685-1.240)	0.589	46.4%	0.155
	**Age subgroups**			Fixed	1.487 (0.947-2.337)	0.085	36.0%	0.154
	children and adolescent	1	58		0.910 (0.171-4.851)	0.912	–	–
	young adults	4	353		1.000 (0.563-1.775)	1.000	0	0.733
	older adults	2	128		3.726 (1.649-8.419)	0.002	3.9%	0.308
**PFS**	**ALL**	5	736	Random	1.365 (0.694-2.684)	0.368	54.5%	0.066
**EFS**	**ALL**	10	1477	Fixed	0.962 (0.755-1.227)	0.756	0	0.543
	**Continent**			Fixed				
	Asia	3	426		1.087 (0.710-1.664)	0.702	65.5%	0.055
	Africa	1	131		0.720 (0.228-2.277)	0.576	–	–
	Europe	3	543		0.937 (0.677-1.298)	0.697	0	0.400
	South America	4	377		0.816 (0.332-2.005)	0.657	0	0.906
	**Detection method**			Fixed				
	EBER	3	368		1.238 (0.805-1.902)	0.331	0	0.528
	LMP1	2	298		0.731 (0.346-1.548)	0.413	74.9%	0.046
	EBER/LMP1	6	811		0.880 (0.638-1.212)	0.432	0	0.913
	**Data source**			Fixed				
	Univariate	10	1288		1.018 (0.794-1.307)	0.886	0	0.839
	Multivariate	1	189		0.330 (0.112-0.975)	0.045	–	–
	**Data extraction**			Fixed				
	K-M curve	7	687		1.039 (0.595-1.815)	0.893	0	0.881
	Direct	4	790		0.945 (0.721-1.238)	0.682	53.2%	0.093
**DSS**	**ALL**	8	2061	Fixed	2.312 (1.799-2.972)	<0.001	26%	0.221
	**Continent**							
	Asia	2	483		2.120 (1.315-3.418)	0.002	71.7%	0.060
	Europe	3	656		2.629 (1.738-3.977)	<0.001	0	0.999
	North America	3	922		2.165 (1.420-3.300)	<0.001	62.5%	0.069
	**Detection method**			Fixed				
	EBER	3	920		2.334 (1.644-3.313)	<0.001	48.3%	0.144
	LMP1	1	102		2.660 (1.099-6.436)	0.030	–	–
	EBER/LMP1	4	1039		2.223 (1.499-3.297)	<0.001	45.0%	0.142
	**Data source**			Fixed				
	Univariate	7	1902		2.189 (1.686-2.844)	<0.001	18.1%	0.292
	Multivariate	1	159		4.396 (1.792-10.785)	0.001	–	–
	**Data extraction**			Fixed				
	K-M curve	5	1542		2.504 (1.859-3.373)	<0.001	0	0.978
	Direct	3	519		1.902 (1.192-3.033)	0.007	75.2%	0.018
	**Age subgroups**			Fixed	2.094 (1.506-2.912)	<0.001	1.2%	0.415
	children and adolescent	1	36		0.180 (0.020-1.660)	0.130	–	–
	young adults	2	845		1.744 (0.736-4.131)	0.206	0	0.965
	older adults	4	501		2.308 (1.607-3.312)	<0.001	0	0.817

EBV, Epstein–Barr virus; HL, Hodgkin’s lymphoma; cHL, classical Hodgkin lymphoma; HR, hazard ratios, EBER, Epstein–Barr virus-encoded small RNA; LMP1, latent membrane protein-1; OS, Overall survival; FFS, Failure-free survival; PFS, Progression-free survival; EFS, Event-free survival; DSS, Disease-specific survival; K-M curve, Kaplan-Meier curve.

#### Failure-free survival

**Figure 3:**
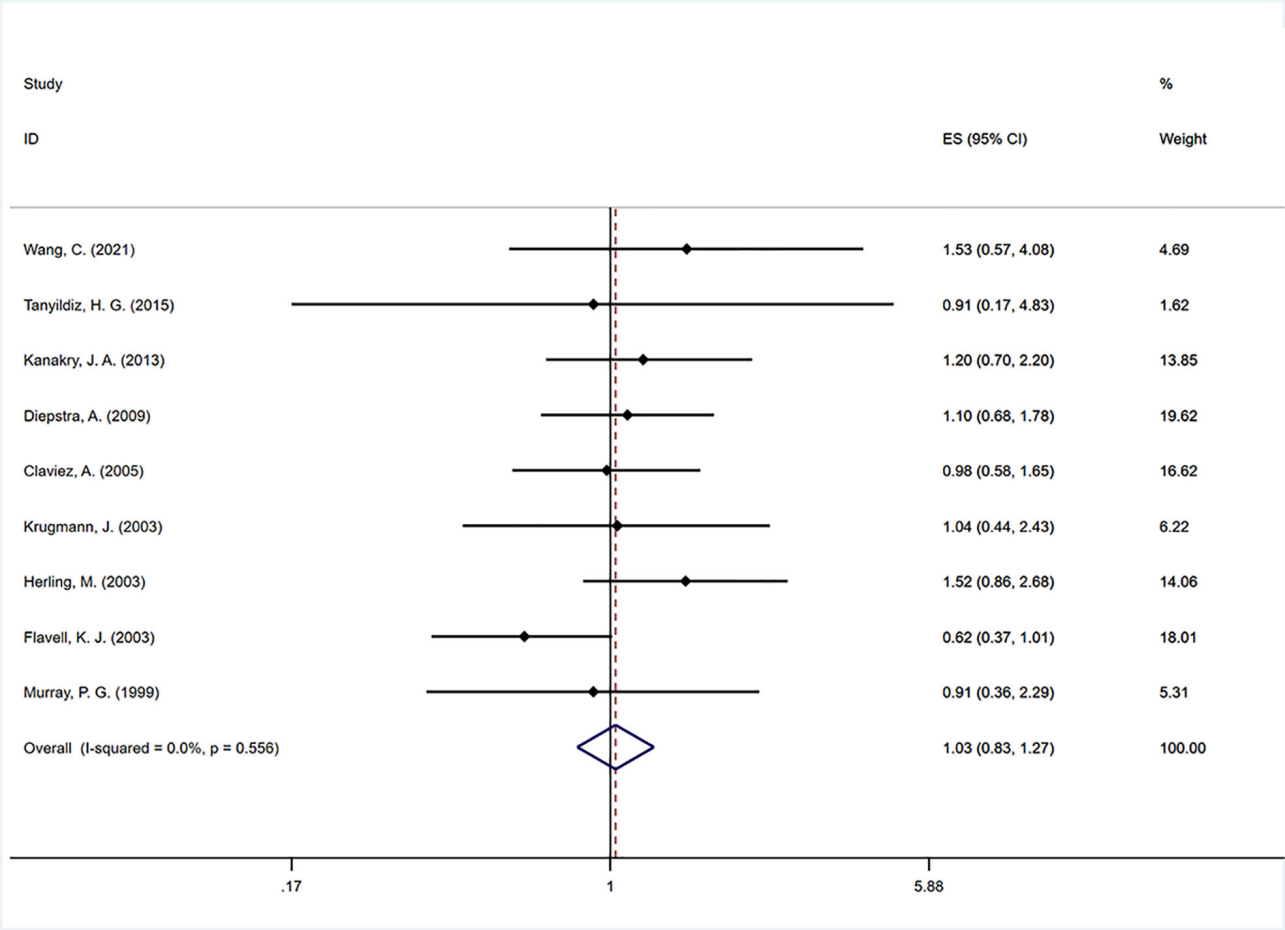
Forest plot of the hazard rations for failure free survival (FFS) between patients with EBV-positive and EBV-negative HL.

Nine studies (11, 21–24, 32, 37, 41, 53) were included to analyze the impact of EBV infection on FFS. The pooled estimate showed no significant association between EBV positivity and FFS (HR=1.030, 95% CI: 0.832–1.274, P=0.788; **Figure 3**). No significant heterogeneity was found across the studies (I^2 =^ 0, P=0.556). In the subgroup analysis, we found that EBV positivity was strongly associated with poorer FFS (HR=3.726, 95% CI: 1.649–8.419, P=0.002) in older adults. In addition, the prognostic results of the three subgroups grouped according to detection method, data source and data extraction were similar, and all had no effect on FFS (**Table 2**).

#### Progression-free survival

**Figure 4:**
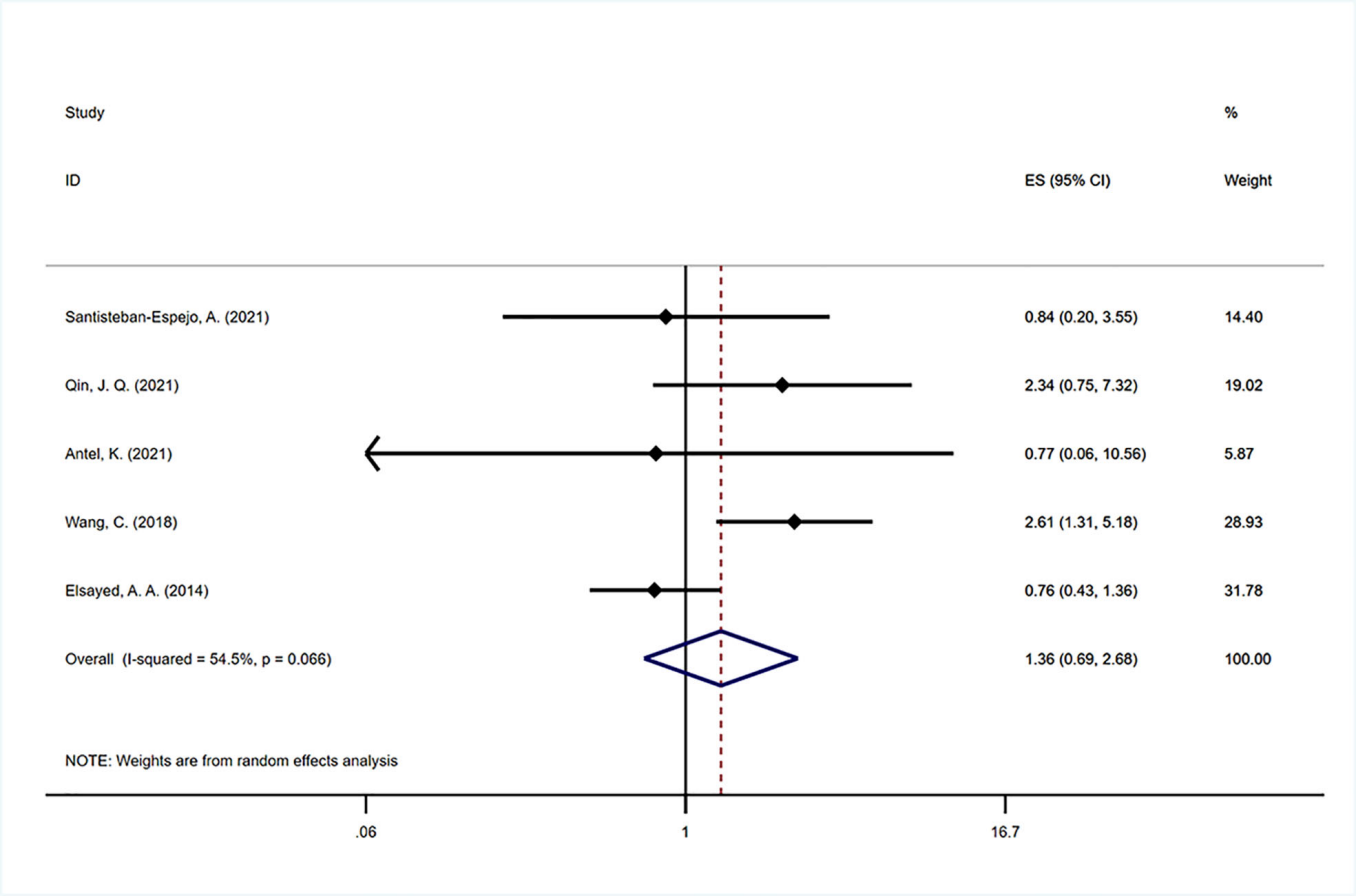
Forest plot of the hazard rations for progression free survival (PFS) between patients with EBV-positive and EBV-negative HL.

Five studies (39, 47, 50–52) were included to analyze the impact of EBV infection on PFS. There was significant between-study heterogeneity (I^2 =^ 54.5%, P=0.066), and the pooled estimate by the random-effects model showed that no significant association was found between EBV positivity and PFS (HR=1.302, 95% CI: 0.881–1.926, P=0.186; **Figure 4**). Due to the lower number of analyzable studies, subgroup analysis was not performed for PFS.

#### Event-free survival

**Figure 5:**
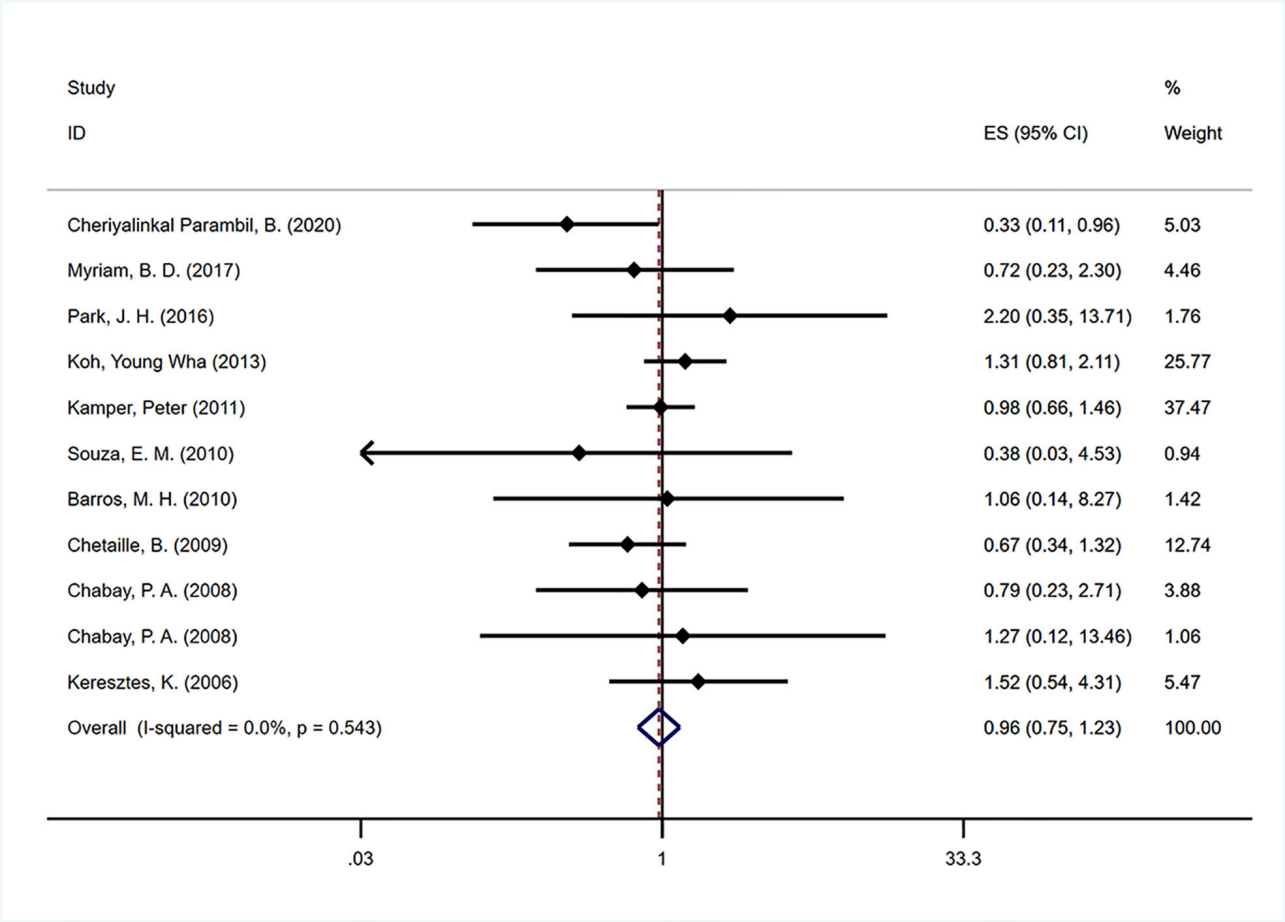
Forest plot of the hazard rations for event free survival (EFS) between patients with EBV-positive and EBV-negative HL.

Ten studies (29–31, 33–35, 38, 42, 44, 48) were included to analyze the impact of EBV infection on EFS. The pooled estimate showed that no significant association was found between EBV positivity and EFS (HR=0.962, 95% CI: 0.755-1.227, P=0.756; **Figure 5**). No significant heterogeneity was found across the studies (I^2 =^ 0, P=0.543). The prognostic effects were similar between the four predefined subgroups according to continent, detection method, data source and extraction (**Table 2**).

#### Disease-specific survival

**Figure 6:**
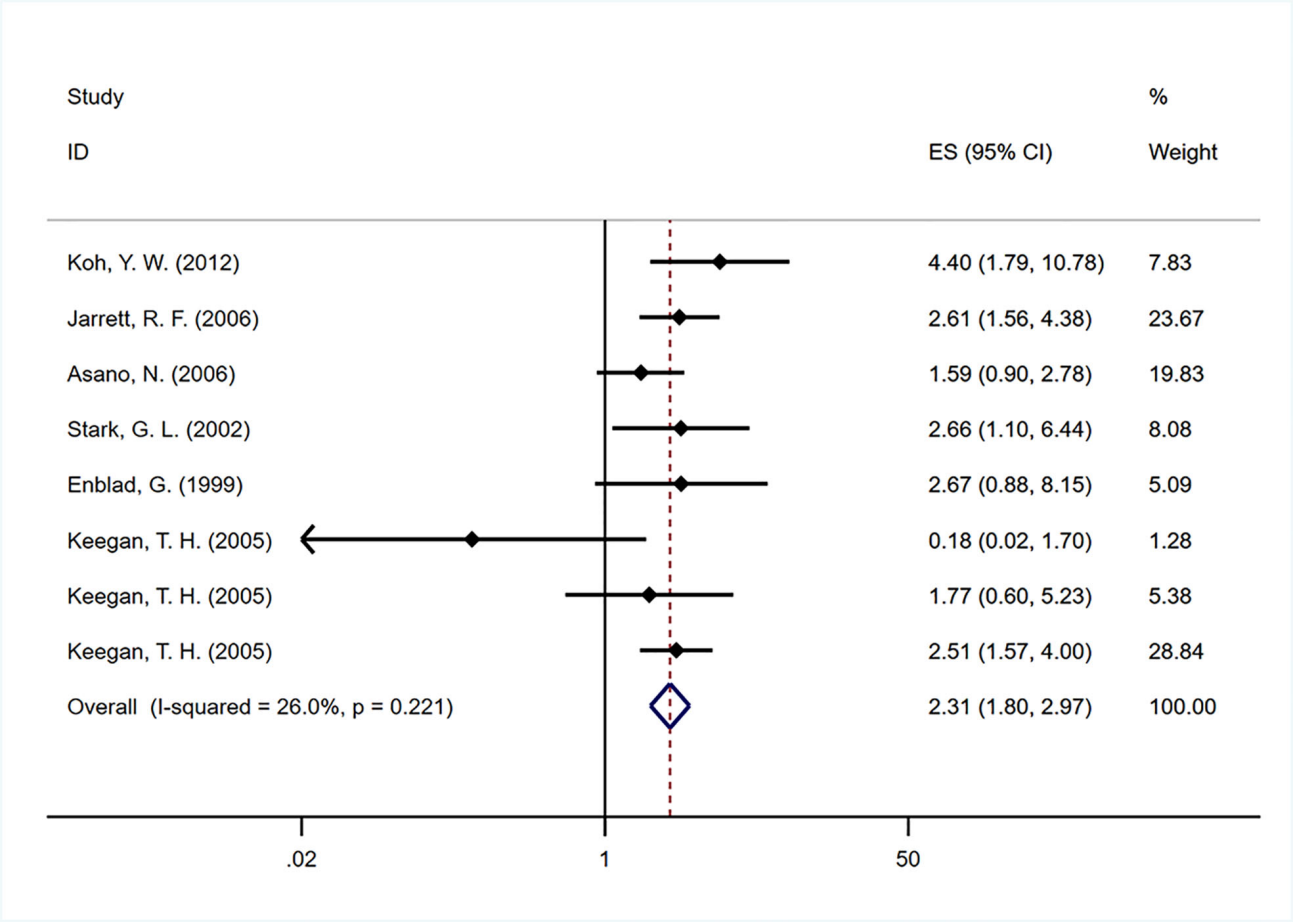
Forest plot of the hazard rations for disease-specific survival (DSS) between patients with EBV-positive and EBV-negative HL.

Six studies (15, 20, 25, 26, 28, 36) were included to analyze the impact of EBV infection on DSS. The pooled HR of 2.312 (95% CI: 1.799-2.972) was calculated on the basis of a fixed-effects model (**Figure 6**), which showed a worse DSS among EBV-positive patients than EBV-negative patients. In subgroup analysis, a fixed-effects model was used for the age subgroup meta-analysis due to the heterogeneity among studies (I^2 =^ 1.2, P=0.415). Interestingly, EBV-positive older adults had poorer DSS (HR=2.308, 95% CI: 1.607-3.312; P<0.001) than EBV-negative adults, whereas studies involving children, adolescents and young adults yielded no association between EBV infection and DSS (**Table 2**).

### Sensitivity analysis and publication bias

We conducted a sensitivity analysis of the association between EBV infection and survival outcomes and demonstrated that the results were robust after omitting any of the included studies (**Figure S1**).

Funnel plots with Begg’s test and Egger’s test were used to assess publication bias, and no evidence of bias was found in our meta-analysis of the selected studies. The p values were all >0.05, and details of the survival outcome publication bias can be seen in **Figure S2**.

## Discussion

The prognostic significance of EBV infection in HL patients remains controversial. Here, we conducted a meta-analysis involving 9570 patients from 42 studies to systematically explore the prognostic value of EBV infection in HL. Our results demonstrated that EBV positivity predicted short DSS and OS, but it had no significant effect on FFS, PFS or EFS. Moreover, subgroup analysis showed that in children and adolescent HL patients, EBV positivity allowed some survival advantage compared with the outcomes of EBV-negative patients, although the difference was not statistically significant. In contrast, EBV-positive elderly patients with HL have strongly poorer survival outcomes than EBV-negative patients.

To our knowledge, this study is an update of two meta-analyses published in 2014 (62) and 2015 (63) on EBV infection and HL OS. Chen, Y. P. et al. (63) found no significant association between EBV infection and overall survival, but their age-specific subgroup analyses showed that OS was significantly shorter when patients’ median/mean age was ≥40 years. In addition, they found that EBV positivity had a tendency for worse OS in patients in Europe and North America. Similar to the findings of Chen and colleagues, a meta-analysis by Lee, J. H. et al. (62) also failed to reveal an association between EBV infection status and cHL patient survival. The reason for the different results between the two meta-analyses above and ours may be that we included more studies published in recent years; moreover, the type of disease was not limited to cHL, and the detection of EBV infection was not restricted to LMP1.

For the survival endpoints of OS and DSS, our results are in line with 7 studies (20, 28, 36, 39, 46, 47, 49), and other reports describing clinical outcomes in relation to EBV status are conflicting. Many studies have not demonstrated that EBV status has an impact on prognosis (35, 38, 40–42, 44, 45, 51–54), whereas some studies have shown that EBV-positive status is associated with a favorable clinical outcome (14, 16, 17, 23, 48, 50). The discrepancies observed in these studies were generally due to the heterogeneous nature of the disease and the selection bias of study subjects, age groups, EBV detection, treatment regimens and the different outcome measures used. Additionally, since the distribution of EBV varies widely in the population, it may reflect racial or ethnic differences. In fact, our subgroup analyses showed that the HR of OS and DSS was not influenced by whether nodular lymphocytic predominant Hodgkin’s lymphoma (NLPHL) patients were excluded, whether the data were extracted from the KM curve and different detection methods. Only the pooled HR from Africa had a tendency to improve OS; interestingly, two of the three included studies enrolled populations younger than 50 years old (16, 50). This was in good agreement with the findings obtained from our subgroup analysis of different age groups. The effect of EBV status on OS and DSS is age dependent, and older adult patients with EBV-positive HL had a particularly poor prognosis, which was consistent with the findings of some population-based studies (18, 25, 28, 38, 50). Our study showed better survival trends for children and adolescents, although this trend was not statistically significant. The abovementioned differential effects on outcomes with respect to age and geography may be attributed to the following reason. EBV infection rates in patients with HL were significantly higher in African and South American countries than in other regions, according to an epidemiological survey (62); therefore, children had a relatively high risk of early exposure to a wide range of infectious agents. As LMP1 has antigenicity, LMP1 could activate cytotoxic T lymphocytes (CTLs) more effectively, resulting in a stronger antitumor immune response (64), which may in turn limit disease progression. However, cytotoxic T-cell responses have been observed to decline with age (65, 66), and another possibility is immunosenescence, in which the impaired immune system is unable to respond effectively to viral infection, allowing EBV reactivation and oncogenic transformation (67). In summary, the younger group has a beneficial EBV-specific immune response to the tumor cell population, whereas in older patients, this response may be less effective or other negative prognostic factors may outweigh any beneficial effect EBV may have. For example, elderly patients have poor treatment tolerance, with a subset unable to tolerate enough chemotherapy or combined radiotherapy and chemotherapy. Furthermore, elderly patients may have had complications that harmed their chances of survival (68).

Herling et al. (22) considered that selection of the study endpoint may be an important factor affecting EBV status and prognosis, and compared to OS and DSS, FFS is a better survival endpoint. OS and DSS are both affected by salvage management after relapse, which was not even mentioned in most studies. Meanwhile, the frequency of disease-unrelated deaths is relatively high in the elderly population, and the natural limitation of life expectancy, these deaths may obscure disease effects in older adult patients. Our meta-analysis concluded that EBV infection status did not affect FFS in the entire population, which is consistent with the results of many previous studies (11, 21, 22, 24, 32, 37, 41, 53). However, Wang, C. et al. (53) and Diepstra, A (32). illustrated that the prognosis was significantly worse for EBV-positive than EBV-negative patients when patients were older than 50 years. Because our age subgroup analysis included the same two papers as well, the results were similar. Only one article (23) reported that EBV-positive patients have a longer FFS than EBV-negative patients; this is the sole study from Australia, which contradicts my results and is probably related to geographical differences.

As with FFS, EFS and PFS were also unaffected by EBV infection status. The ending endpoint was PFS in a small number of articles (23, 39, 47, 50–52), and we did not perform further subgroup analyses. A child-based study from India (48) showed that EBV-positive children have longer EFS than EBV-negative children, which contradicted our finding and may be explained by the high prevalence of EBV infection in Indian children and the greater chemotherapy and radiotherapy sensitivity of infected tumor cells (17).

At present, the mechanism by which EBV acts on HL is still unclear (69). In EBV-positive HL, viral infection of malignant tumor cells is characterized by the consistent expression of three EBV-associated viral proteins (EBNA1, LMP1, and LMP2A) and two noncoding RNAs (EBERs and BARTs) (67), which are believed to play important roles in tumorigenesis, including the regulation of proliferation, metastasis, immune escape, and apoptosis (70, 71). EBNA1 enhanced the activity of the AP‐1 transcription factor, triggering the induction of VEGF and IL‐8 (72); meanwhile, this protein can inhibit the antigenic peptide bound to major histocompatibility complex 1 (MHC-1) to evade recognition by CTL (73). LMP1 stimulates the proliferation of B cells by activating nuclear factor-kappa B (NFκB) and the transcription factor AP-1 (74). Moreover, LMP1 can also immortalize resting B lymphocytes and turn them into latently infected lymphoblastoid cell lines (75, 76). Collectively, these mechanisms may explain why EBV positivity is associated with poor clinical outcomes in HL patients. There are certain limitations that must be considered when interpreting the results of our study. First, there was some heterogeneity across these included articles, and despite the use of subgroup analysis, it was not feasible to explore all of the variability. Treatment regimens are not clearly indicated in some studies, and many articles do not conduct age-stratified analysis. These limitations prevented us from fully tracing the origin of heterogeneity. Second, the quality of published data for our study was relatively low, and most of the included studies were retrospective in design. Third, the age cutoff between children, adults and elderly varied according to the published studies; thus, to include as many studies as possible, 15-18 years old and 45-50 years old were used as a vague distinction dividing patients into children and adolescents, young adults and older adults. To obtain more meaningful results, more research involving the unified age cutoff is needed. Finally, because this study was limited to studies published in English, publication bias cannot be ruled out. The prevalence of EBV is higher in developing countries, but our study embraces only a small number of studies in Africa and South America. In addition, although some studies have shown that EBV-DNA can be used as a prognostic marker for EBV-associated HL, the choice of compartments of peripheral blood and cut-off copies of EBV-DNA is different in various studies (37, 51, 77, 78). Given the different criteria in the related original studies, we did not include the studies of using PCR method to detect EBV infection.

## Conclusion

Our findings suggest that EBV-positive status is associated with poor OS and DSS in HL patients. EBV infection should therefore be considered a valuable prognostic marker and risk-stratifying factor in HL, especially in older patients. More studies in the future should include a larger number of children and young adults to investigate the combined effects of age and EBV status with other prognostic factors to improve the therapeutic applicability of these findings.

## Data availability statement

The original contributions presented in the study are included in the article/**Supplementary Material**. Further inquiries can be directed to the corresponding author.

## Author contributions

JH and YJ formulated the research questions and designed the study; JH, XZ, and HT conducted the literature search, selected the articles, and extracted the data; JH and HT analyzed the data and drafted the manuscript; and YJ critically revised the article. All authors contributed to the article and approved the submitted version.

## Funding

This study was supported by the 1·3·5 project for disciplines of excellence–Clinical Research Incubation Project, West China Hospital, Sichuan University (grant number 2020HXFX020).

## Acknowledgments

Thanks to the authors of all the included articles that were used as data sources for this article.

## Conflict of interest

The authors declare that the research was conducted in the absence of any commercial or financial relationships that could be construed as a potential conflict of interest.

## Publisher’s note

All claims expressed in this article are solely those of the authors and do not necessarily represent those of their affiliated organizations, or those of the publisher, the editors and the reviewers. Any product that may be evaluated in this article, or claim that may be made by its manufacturer, is not guaranteed or endorsed by the publisher.
