# Supplementary material for: The prognostic value of Epstein−Barr virus infection in Hodgkin lymphoma: A systematic review and meta-analysis
Source: Front Oncol. 2022 Oct 27;12:1034398. doi: 10.3389/fonc.2022.1034398 (PMC9648611; doi:10.3389/fonc.2022.1034398)

Supplementary materials

Table S1 Definitions of survival endpoints

Table S2 Quality assessment of eligible studies using the Newcastle-Ottawa Scale (NOS)

Fig S1 Sensitivity analysis (a) Overall survival; (b) Failure free survival; (c) Event free survival; (d) Disease-specific survival; (e) Progression free survival

Fig S2 Publication bias (a) Overall survival; (b) Failure free survival; (c) Event free survival; (d) Disease-specific survival; (e) Progression free survival

Table S1 ***Definitions of survival endpoints***

**Endpoints**	**Definition**
Overall survival	the date of diagnosis to death from any cause
Event-free survival	the date of diagnosis to any treatment failure including lymphoma progression, or discontinuation of treatment for any reason including death
Progression-free survival	the date of diagnosis to relapse, progression, or death from any cause
Disease-specific survival	the date of diagnosis until the date of death as a result of a lymphoma-related cause
Failure-free survival	the date of start of treatment to the date of treatment failure (progression or relapse) or death from HL, whichever was first.

Table S2 ***Quality assessment of eligible studies using the Newcastle-Ottawa Scale (NOS)***

		Selection				Comparability	Outcome			
First Author	Year	Representativeness of the exposed cohort	Selection of the nonexposed cohort	Assessment of exposure	Demonstration that outcome of interest was not present at start of study	Comparability of cohorts on the basis of the design or analysis	Ascertainment of outcome	Long enough follow-up for outcomes to occur	Adequacy of follow-up of cohorts	Overall score
Yang, L. Q.	2022	☆	☆	☆	☆	0	☆	☆	☆	7
Wang, C.	2021	☆	☆	☆	☆	☆	☆	☆	☆	8
Santisteban-Espejo, A.	2021	☆	☆	☆	☆	0	☆	☆	☆	7
Qin, J. Q.	2021	☆	☆	☆	☆	☆	☆	☆	☆	8
Antel, K.	2021	☆	☆	☆	☆	☆	☆	☆	☆	8
Werner, L.	2020	☆	☆	☆	☆	☆	☆	☆	☆	8
Cheriyalinkal Parambil, B.	2020	☆	☆	☆	☆	☆	☆	☆	0	7
Wang, C.	2018	☆	☆	☆	☆	☆	☆	☆	☆	8
Koh, Y. W.	2018	☆	☆	☆	☆	☆	☆	☆	☆	8
Hollander, P.	2018	☆	☆	☆	☆	☆	☆	☆	☆	8
Myriam, B. D.	2017	☆	☆	☆	☆	☆	☆	☆	☆	8
Chang, K. C.	2017	☆	☆	☆	☆	☆	☆	☆	☆	8
Park, J. H.	2016	☆	☆	☆	☆	☆	☆	☆	☆	8
Tanyildiz, H. G.	2015	☆	☆	☆	☆	☆	☆	☆	☆	8
Paydas, S.	2015	☆	☆	☆	☆	0	☆	☆	☆	7
Elsayed, A. A.	2014	☆	☆	☆	☆	☆☆	☆	☆	☆	9
Koh, Young Wha	2013	☆	☆	☆	☆	☆	☆	☆	☆	8
Kanakry, J. A.	2013	☆	☆	☆	☆	☆	☆	☆	☆	8
Koh, Y. W.	2012	☆	☆	☆	☆	☆	☆	☆	☆	8
Kamper, Peter	2011	☆	☆	☆	☆	☆	☆	☆	☆	7

Souza, E. M.	2010	☆	☆	☆	☆	☆	☆	☆	☆	8
Barros, M. H.	2010	☆	☆	☆	☆	☆	☆	☆	0	7
Diepstra, A.	2009	☆	☆	☆	☆	☆	☆	☆	☆	8
Chetaille, B.	2009	☆	☆	☆	☆	☆	☆	☆	0	7
Chabay, P. A.	2008	☆	☆	☆	☆	☆	☆	☆	☆	8
Keresztes, K.	2006	☆	☆	☆	☆	☆	☆	☆	0	7
Jarrett, R. F.	2006	☆	☆	☆	☆	☆	☆	☆	☆	8
Asano, N.	2006	☆	☆	☆	☆	0	☆	☆	☆	7
Al-Kuraya, K.	2006	☆	☆	☆	☆	0	☆	☆	0	6
Keegan, T. H.	2005	☆	☆	☆	☆	☆	☆	☆	☆	8
Claviez, A.	2005	☆	☆	☆	☆	☆	☆	☆	☆	8
Krugmann, J.	2003	☆	☆	☆	☆	☆	☆	☆	☆	8
Herling, M.	2003	☆	☆	☆	☆	☆	☆	☆	☆	8
Flavell, K. J.	2003	☆	☆	☆	☆	0	☆	☆	☆	7
Stark, G. L.	2002	☆	☆	☆	☆	☆	☆	☆	☆	8
Glavina-Durdov, M.	2001	☆	☆	☆	☆	☆	☆	0	☆	7
Clarke, C. A.	2001	☆	☆	☆	☆	☆	☆	☆	0	7
Naresh, K. N.	2000	☆	☆	☆	☆	0	☆	☆	0	6
Engel, M.	2000	☆	☆	☆	☆	☆	☆	0	☆	7
Murray, P. G.	1999	☆	☆	☆	☆	☆	☆	0	0	6
Enblad, G.	1999	☆	☆	☆	☆	0	☆	☆	☆	7
Morente, M. M.	1997	☆	☆	☆	☆	☆	☆	☆	☆	8

Fig S1(a)

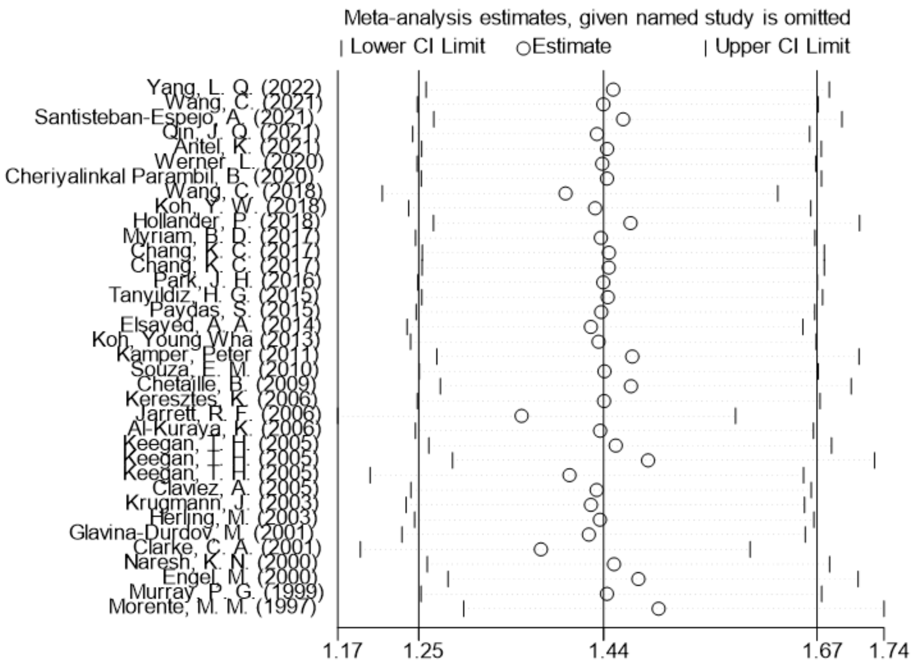

Fig S1(b)

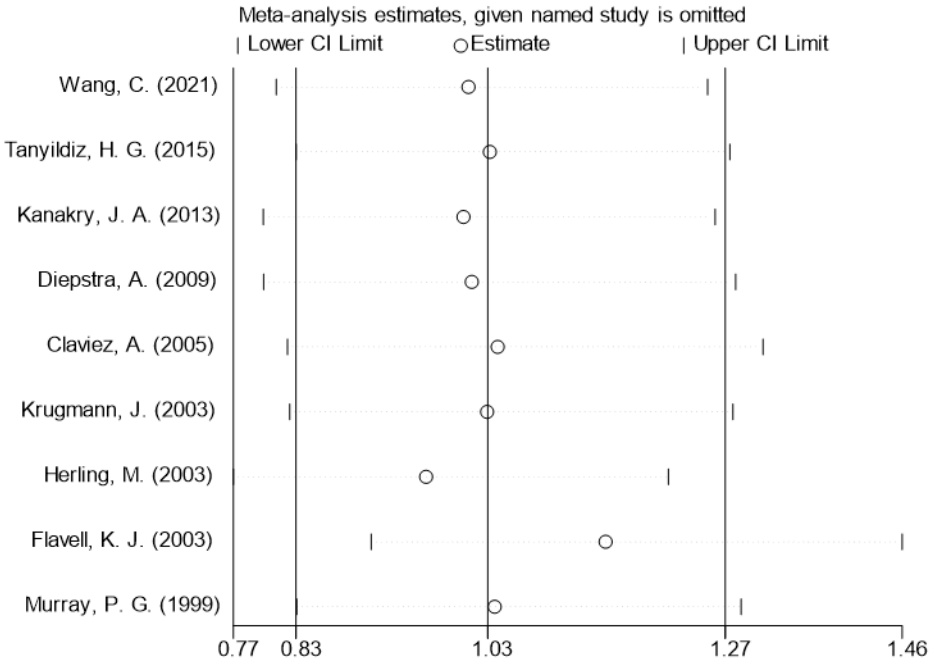

Fig S1(c)

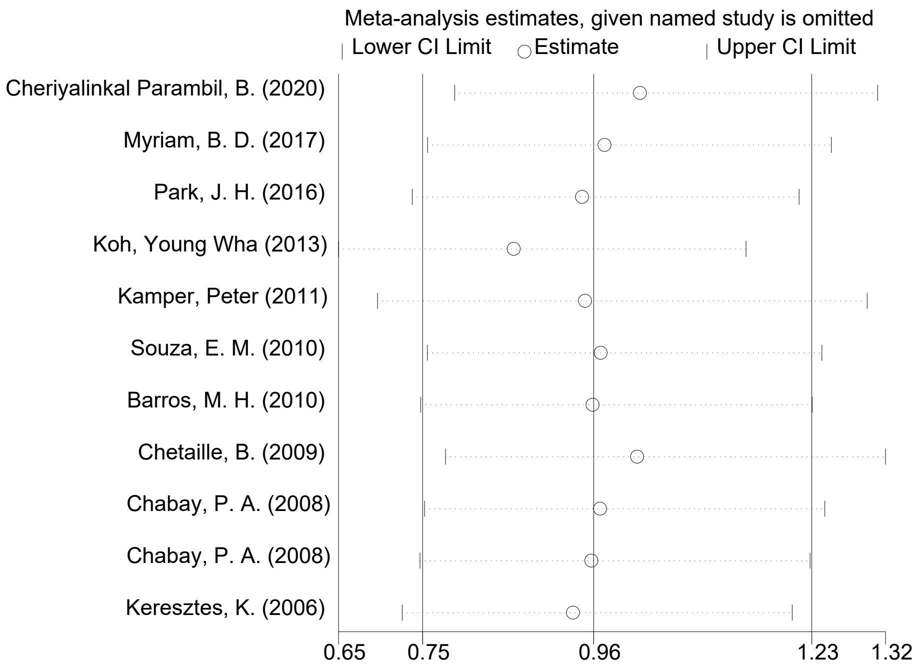

Fig S1(d)

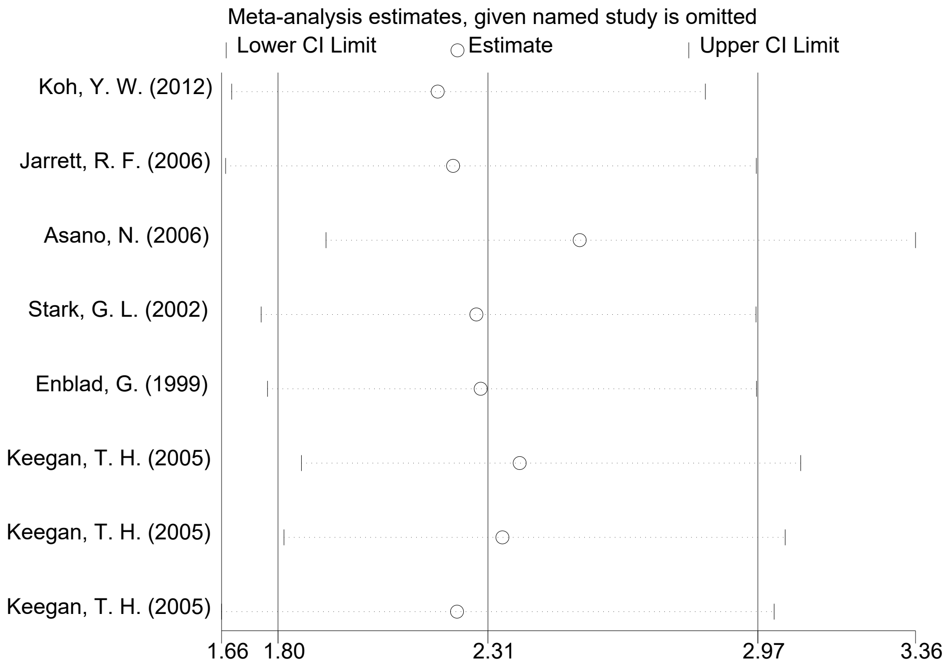

Fig S1(e)

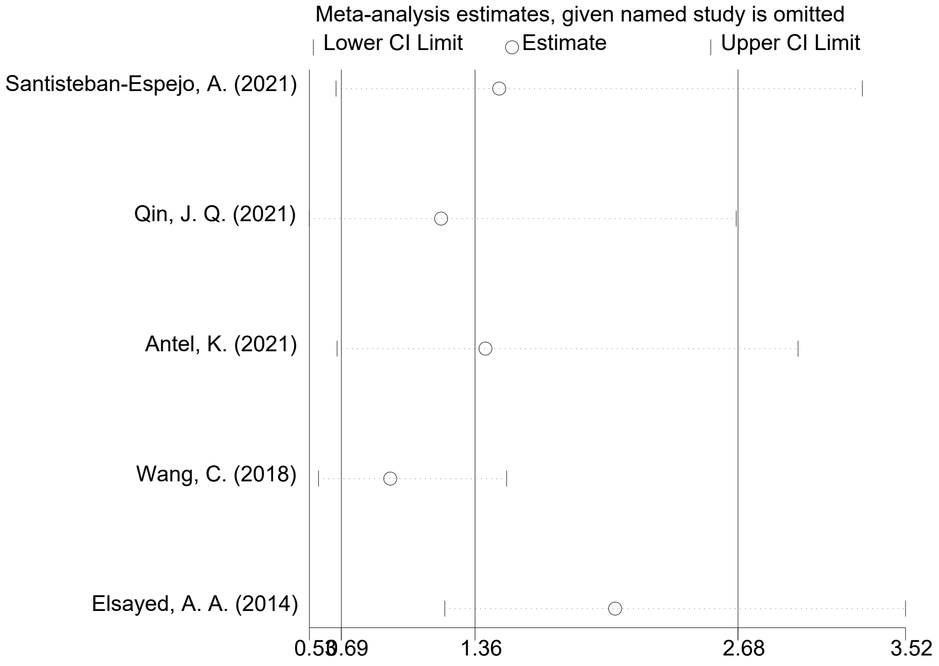

Meta-analysis estimates, given named study is omitted
Lower CI Limit
Estimate
Upper CI Limit
Santisteban-Espejo, A. (2021)
Qin, J. Q. (2021)
Antel, K. (2021)
Wang, C. (2018)
Elsayed, A. A. (2014)
0.69
1.36
2.68
3.52

Fig S2(a)

OS Begg's Test: p=0.406

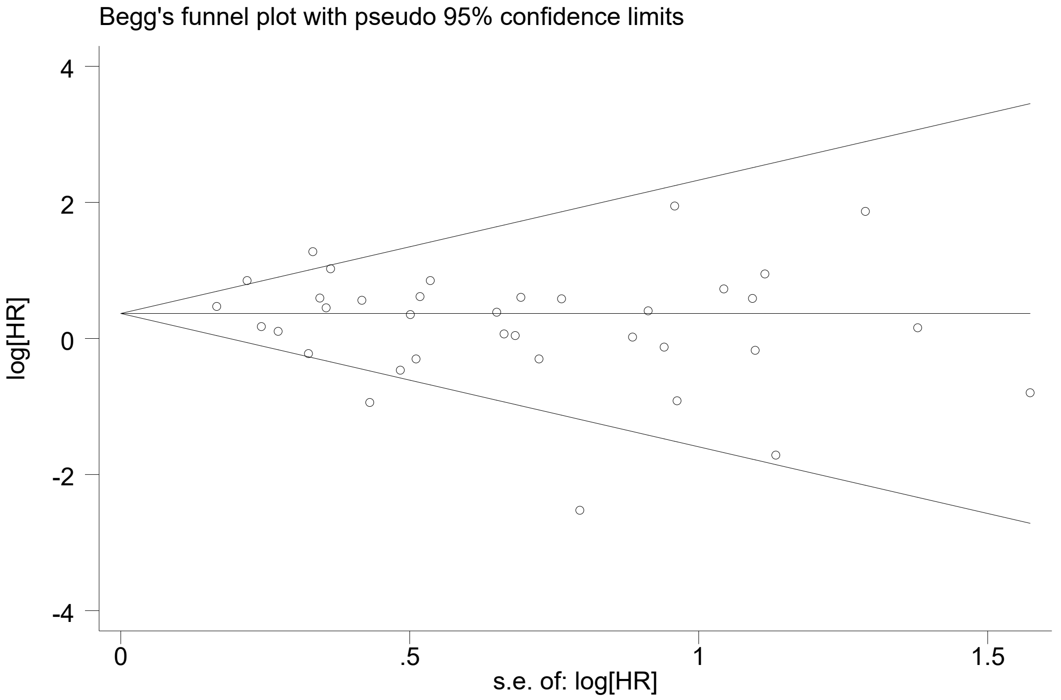

OS Egger's test: p=0.162

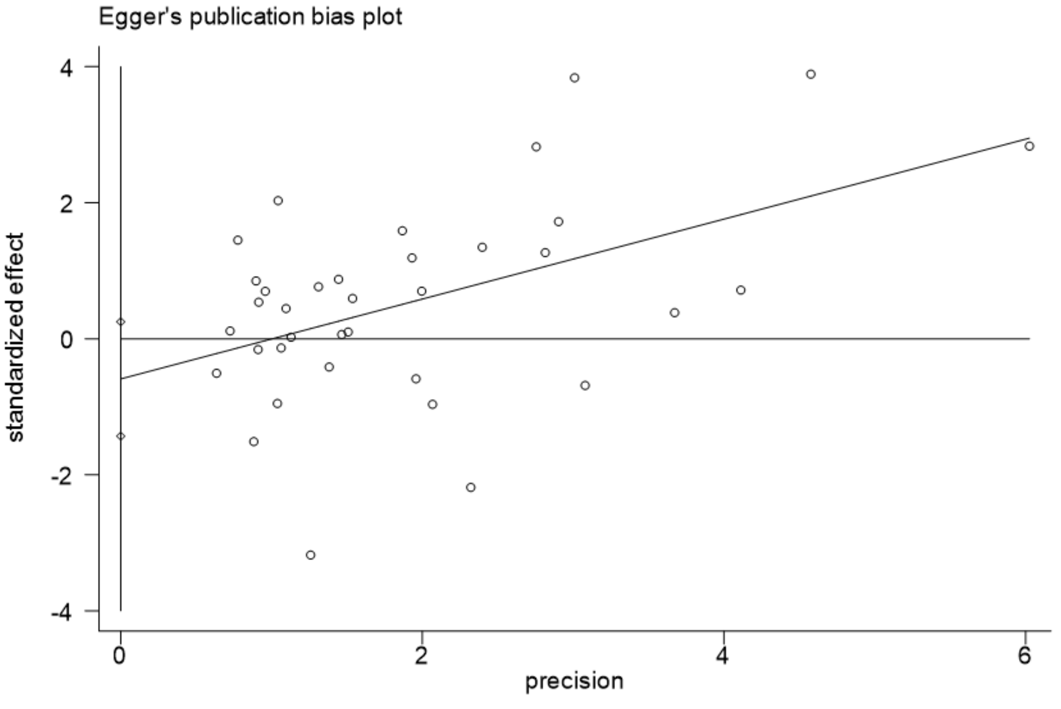

Fig S2(b)

FFS Begg's test: p=0.917

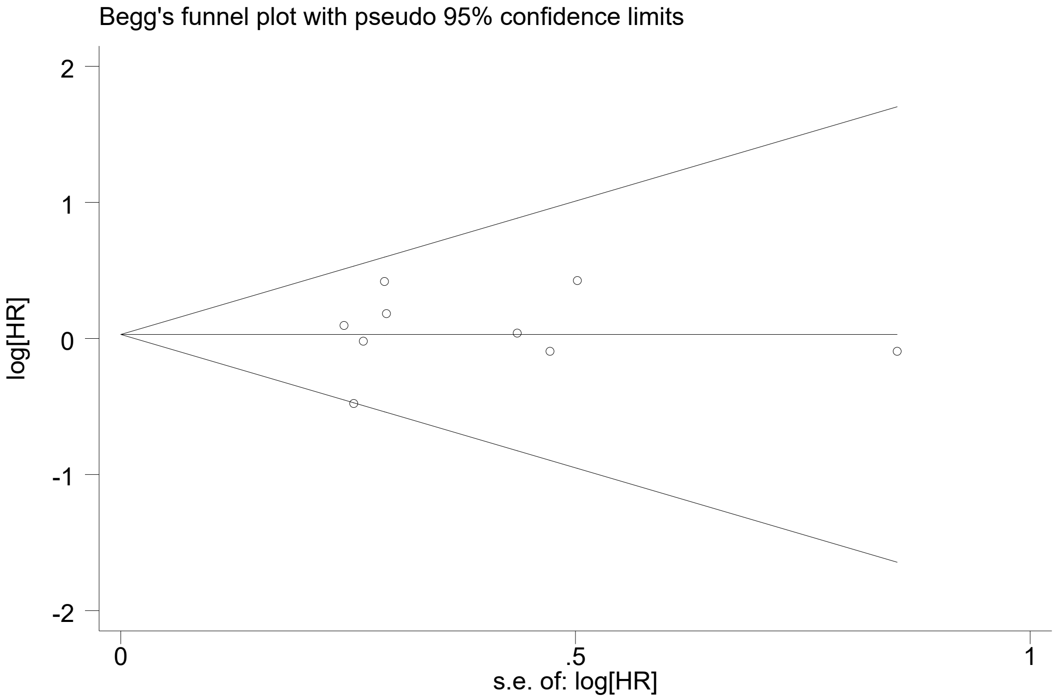

FFS Egger's test: p=0.696

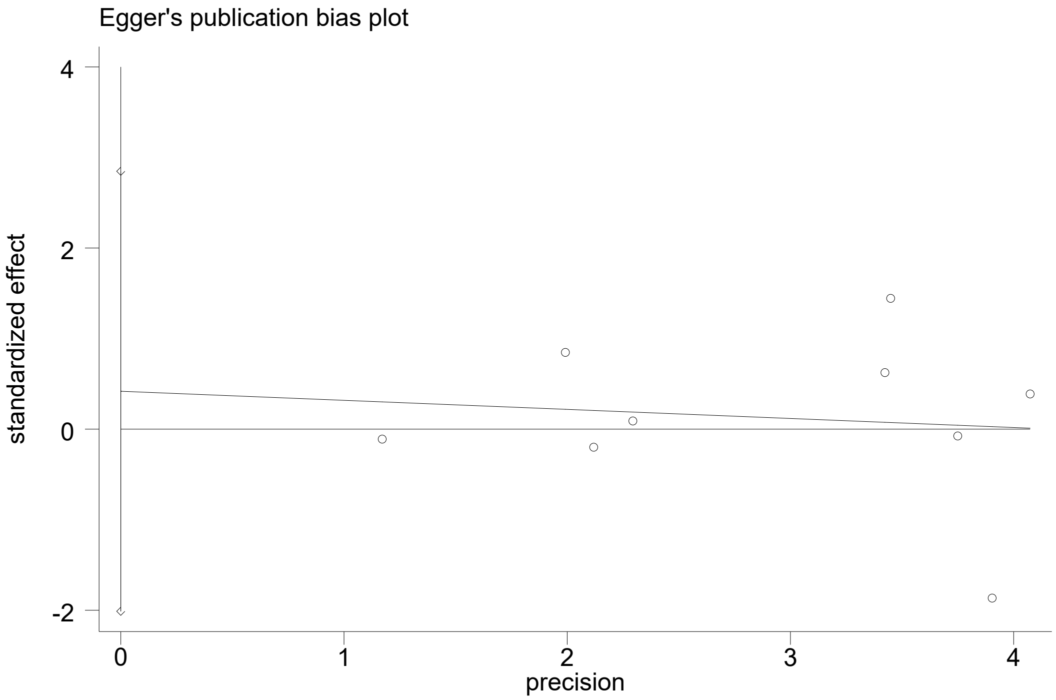

Fig S2(c)

EFS Begg's test: p=0.876

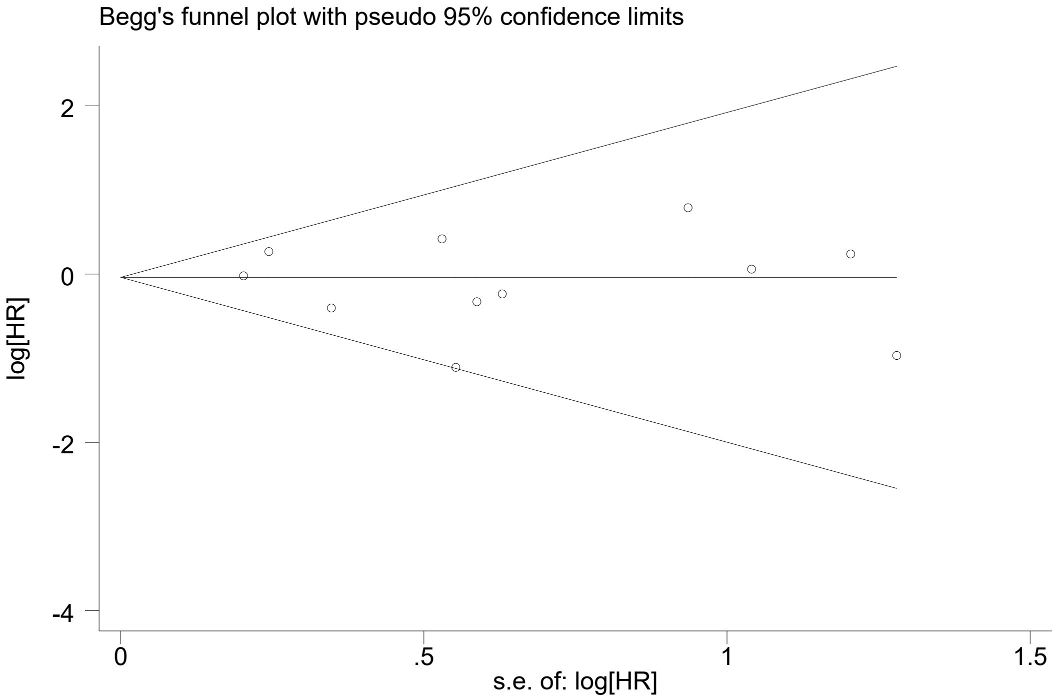

EFS Egger's test: p=0.540

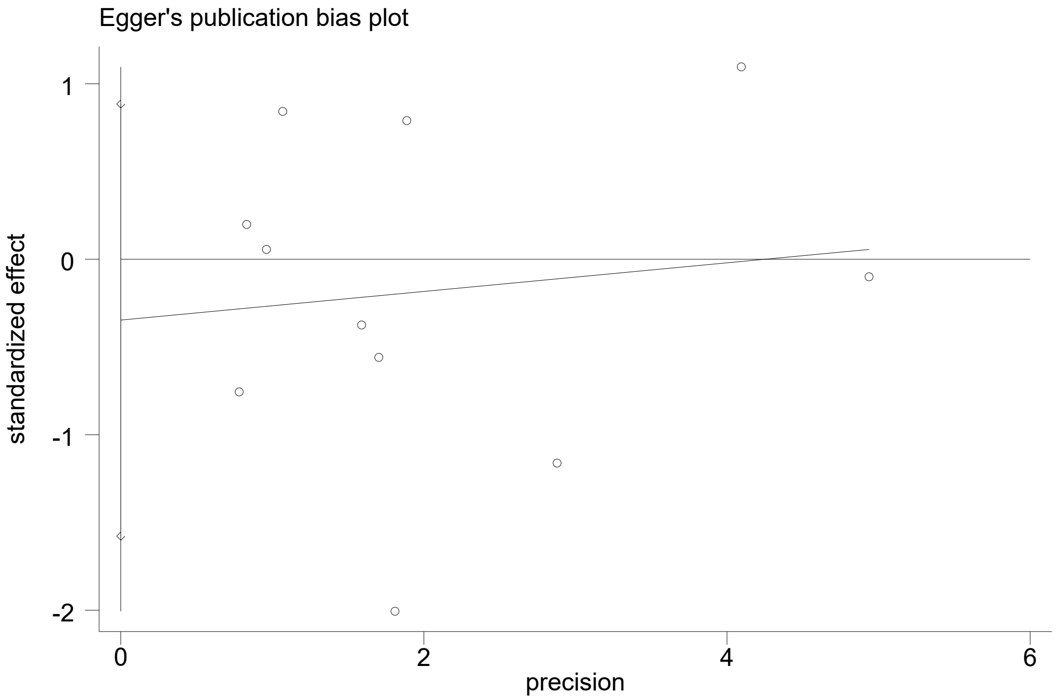

Fig S2(d)

DSS Begg's test: p=0.266

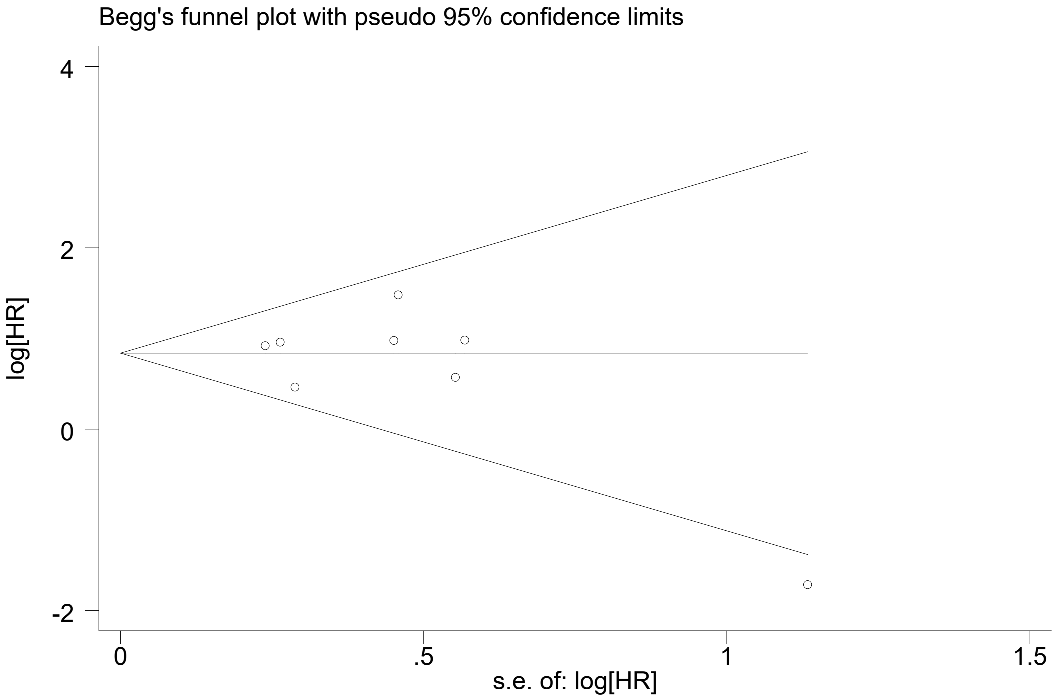

EFS Egger's test: p=0.363

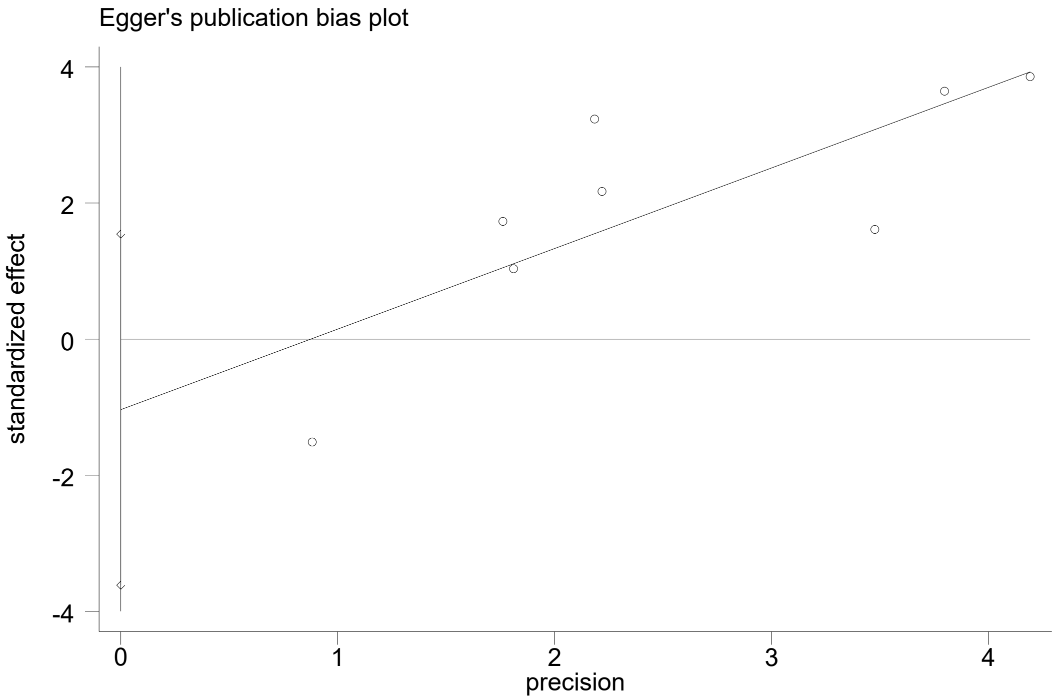

Fig S2(e)

PFS Begg's test: p=1.000

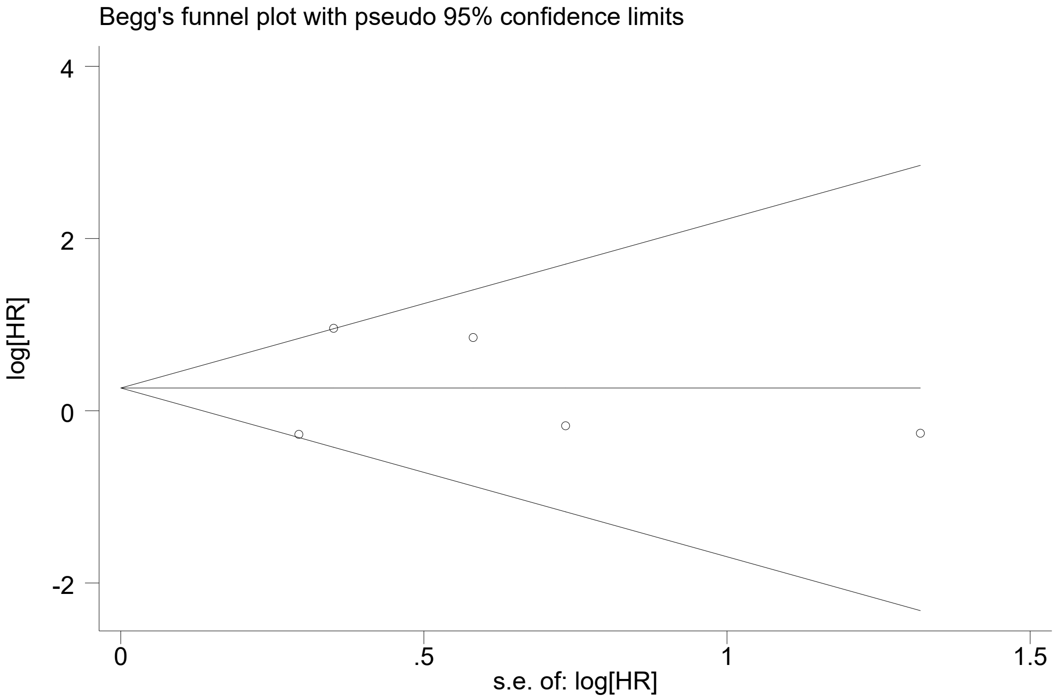

PFS Egger's test: p=0.932

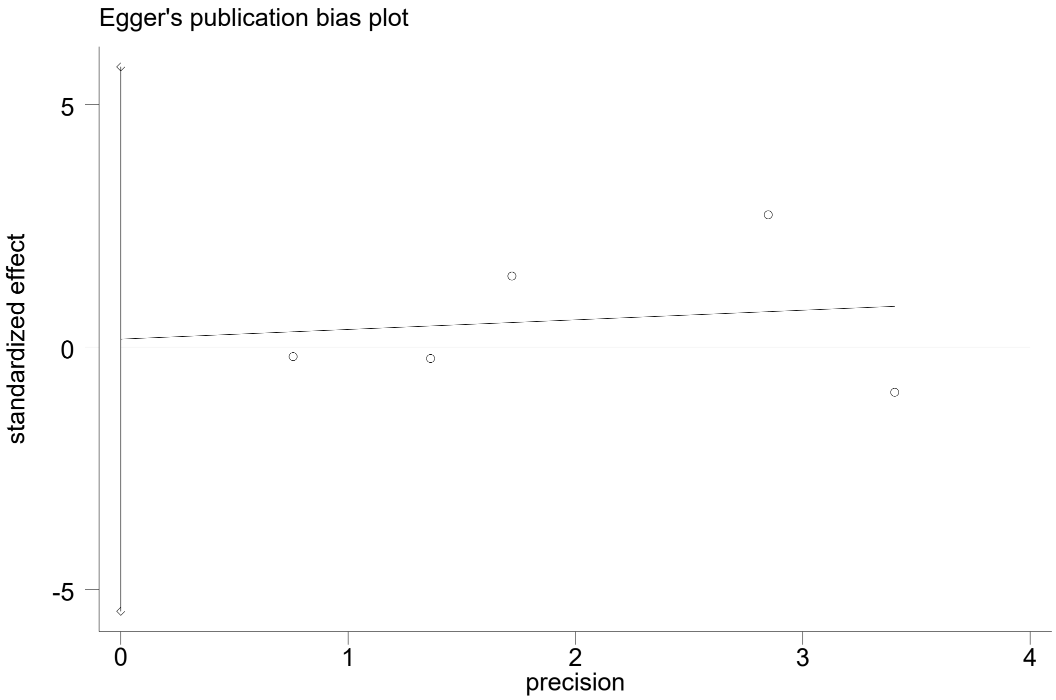

Supplement: Supplementary file 1 [file DataSheet_1.pdf]
